# Immunotherapy in NK/T-Cell Lymphoma: Mechanisms, Clinical Evidence, Resistance, and Emerging Multimodal Strategies

**DOI:** 10.3390/cancers18091358

**Published:** 2026-04-24

**Authors:** Qihao Zhang, Xin Wang

**Affiliations:** Department of Hematology, The First Affiliated Hospital of Chongqing Medical University, Chongqing 400016, China

**Keywords:** NK/T-cell lymphoma, Epstein–Barr virus, tumor immune microenvironment, immunotherapy, PD-1/PD-L1 inhibitors

## Abstract

Natural killer/T-cell lymphoma (NKTCL) is a rare and aggressive Epstein–Barr virus (EBV)-associated lymphoma characterized by intrinsic chemoresistance and an immunosuppressive tumor immune microenvironment (TIME). EBV-driven immune dysregulation provides a biological rationale for immunotherapy. This review summarizes current advances in immunotherapeutic strategies for NKTCL, integrating molecular mechanisms, clinical evidence, and resistance mechanisms within the context of TIME remodeling and immune reprogramming. We synthesize evidence from clinical trials, translational studies, and preclinical investigations evaluating immune checkpoint inhibitors, antibody-based therapies, adoptive cellular therapies, immune engagers, EBV-directed immunotherapies, and multimodal combination strategies in NKTCL. Among these strategies, PD-1/PD-L1 inhibitors are the most extensively studied immunotherapies in NKTCL and demonstrate clinically meaningful activity across different clinical settings. However, therapeutic responses remain heterogeneous, and primary or acquired resistance is common, driven by EBV-associated immune suppression, defective antigen presentation, metabolic reprogramming, and multi-checkpoint co-expression. Beyond immune checkpoint blockade, emerging approaches—including dual-checkpoint inhibition, epigenetic and metabolic combinations, antibody–drug conjugates, EBV-specific cytotoxic T lymphocytes, chimeric antigen receptor (CAR)-based platforms, immune engagers, and EBV vaccines—have shown encouraging signals in early-phase studies. Increasing evidence also supports multimodal strategies integrating immunotherapy with radiotherapy and other immune-modulatory interventions to enhance immune reprogramming and improve response durability. Overall, immunotherapy has substantially expanded the therapeutic landscape of NKTCL but remains constrained by complex EBV–TIME interactions and interpatient heterogeneity. Future progress will rely on biologically informed patient stratification, rational multimodal combination strategies, and integration of innovative immune platforms to establish a durable, immune-reprogramming-centered treatment paradigm for EBV-driven NKTCL.

## Simple Summary

Natural killer/T-cell lymphoma is a rare but highly aggressive cancer that is closely linked to infection with the Epstein–Barr virus. Standard chemotherapy often has limited efficacy for this disease, and many patients relapse or experience severe side effects. In recent years, immunotherapy-based treatments have expanded treatment options for this disease. This review summarizes current and emerging immunotherapy-based treatment strategies with potential relevance for this disease and highlights how combining different therapeutic approaches may help improve and prolong treatment responses. These approaches include various forms of immunotherapy and strategies that combine immunotherapy with radiotherapy or other treatments that modify the tumor microenvironment. By integrating mechanistic insights with clinical evidence, this review outlines how immunotherapy is reshaping treatment options and suggests future directions toward more durable and personalized therapies for this challenging disease.

## 1. Introduction

Natural killer/T-cell lymphoma (NKTCL), previously termed extranodal NK/T-cell lymphoma, nasal type (ENKTL), is a rare and highly aggressive subtype of non-Hodgkin lymphoma. It is strongly associated with Epstein–Barr virus (EBV) infection and is clinically characterized by predominant extranodal involvement. The disease most commonly arises from natural killer (NK) cells and is characterized by a germline configuration of the T-cell receptor (TCR) genes together with a typical NK-cell immunophenotype (CD2^+^, CD56^+^, cytoplasmic CD3ε^+^, and absence of surface TCR expression). A minority of cases arise from T cells and harbor clonal TCR gene rearrangements. Despite these differences, both entities share highly overlapping clinical and pathological features and are therefore collectively classified as NKTCL [1,2]. NKTCL shows poor responsiveness to conventional anthracycline-based chemotherapy regimens, such as CHOP, primarily owing to high expression of P-glycoprotein (P-gp) in tumor cells, which mediates multidrug resistance [3,4]. In addition, EBV-driven immune evasion mechanisms play a central role in therapeutic resistance. Viral latent membrane proteins, particularly latent membrane protein 1 (LMP1) and latent membrane protein 2A (LMP2A), activate oncogenic signaling pathways and induce programmed death-ligand 1 (PD-L1) upregulation, leading to functional exhaustion of T and NK cells. This process is further reinforced by the accumulation of immunosuppressive components within the tumor immune microenvironment (TIME), including M2-polarized macrophages, regulatory T cells (Tregs), and exhausted effector T cells. At present, non-anthracycline, asparaginase-based chemotherapy regimens—including SMILE [5,6,7], AspaMetDex [8], modified SMILE [9], P-GemOx [10], and DDGP [11]—have become the standard therapeutic backbone and have substantially improved outcomes, particularly in patients with early-stage disease. Nevertheless, these approaches remain limited by considerable treatment-related toxicity, poor long-term survival in advanced-stage disease, and high rates of relapse. Consequently, there is an urgent need for safer and more effective therapeutic strategies.

**Figure 1:**
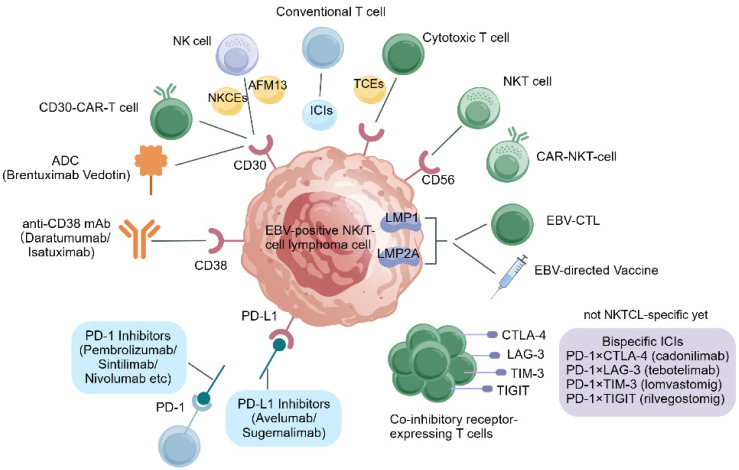
Overview of the immunotherapeutic landscape in NK/T-cell lymphoma. EBV-positive NKTCL is characterized by a profoundly immunosuppressive TIME driven by viral latent proteins such as LMP1 and LMP2A, leading to checkpoint upregulation, impaired antigen presentation, and T/NK-cell exhaustion. This schematic centers on the EBV-infected malignant NK/T cell and outlines the principal and emerging immunotherapeutic strategies for NKTCL, including PD-1/PD-L1 blockade, antibody-based therapies (targeting CD30 and CD38), adoptive cellular therapies (CAR-T, CAR-NKT, EBV-CTLs), immune engagers (TCEs and NKCEs), EBV-directed vaccines, and exploratory bispecific checkpoint-based approaches. The figure emphasizes that durable therapeutic efficacy requires coordinated remodeling of the TIME rather than reliance on single-pathway inhibition. Created with BioGDP.com [12]. Abbreviations: EBV, Epstein–Barr virus; NKTCL, natural killer/T-cell lymphoma; TIME, tumor immune microenvironment; LMP1, latent membrane protein 1; LMP2A, latent membrane protein 2A; PD-1, programmed cell death protein 1; PD-L1, programmed death-ligand 1; CTLA-4, cytotoxic T-lymphocyte-associated protein 4; LAG-3, lymphocyte activation gene-3; TIM-3, T-cell immunoglobulin and mucin-domain containing-3; TIGIT, T cell immunoreceptor with Ig and ITIM domains; ICIs, immune checkpoint inhibitors; CAR-T cells, chimeric antigen receptor T cells; CAR-NKT cells, chimeric antigen receptor natural killer T cells; EBV-CTLs, Epstein–Barr virus-specific cytotoxic T lymphocytes; NKCEs, natural killer cell engagers; TCEs, T-cell engagers.

In recent years, advances in the understanding of TIME have positioned immunotherapy as a promising therapeutic paradigm in NKTCL. The strong association with EBV, the presence of disease-specific antigens, and the distinctive immunoregulatory features of NKTCL provide a compelling biological rationale for immune-based interventions. To date, multiple immunotherapeutic modalities—including immune checkpoint inhibitors (such as anti-PD-1/PD-L1 antibodies), emerging bispecific checkpoint inhibitors, adoptive cellular therapies (including chimeric antigen receptor T cells [CAR-T cells], chimeric antigen receptor natural killer T cells [CAR-NKT cells], and Epstein–Barr virus-specific cytotoxic T lymphocytes [EBV-CTLs]), antibody-based therapies targeting surface antigens such as CD30 and CD38, immune engagers (including T-cell engagers [TCEs] and NK-cell engagers [NKCEs]), as well as EBV-directed vaccines—have demonstrated encouraging activity in preclinical studies and early-phase clinical trials. By modulating the TIME and reinvigorating antitumor immune responses, immunotherapy holds the potential to overcome the limitations of conventional treatments and to improve clinical outcomes in patients with NKTCL. From a clinical perspective, the current role of immunotherapy in NKTCL differs across disease settings. In early-stage disease, immunotherapy is mainly being explored in combination with radiotherapy or pegaspargase-based treatment backbones. In advanced-stage disease, it is increasingly being incorporated into first-line immunochemotherapy regimens, whereas in relapsed or refractory disease, PD-1/PD-L1 blockade currently represents the most clinically established immune-based approach. By contrast, cellular therapies, immune engagers, and EBV-directed vaccines remain largely investigational and are being evaluated in selected translational or early clinical settings. In this review, we comprehensively summarize current advances in immunotherapeutic strategies for NKTCL by integrating their mechanistic basis, clinical evidence, resistance architecture, and translational implications, and we further discuss future directions and remaining challenges. An overview of the immunotherapeutic landscape in NK/T-cell lymphoma, encompassing the major immune-based strategies discussed in this review, is provided in Figure 1.

## 2. Advances in Immune Checkpoint Inhibitors (ICIs) in NKTCL

### 2.1. Mechanisms of EBV-Driven Activation of the PD-1/PD-L1 Axis

As a central mechanism of immune escape in NKTCL, EBV-driven activation of the PD-1/PD-L1 axis provides an important entry point for understanding the immunobiology and therapeutic rationale of this disease. In this context, sustained overexpression of PD-L1 contributes substantially to the establishment of an immunosuppressive tumor immune microenvironment. The upregulation of PD-L1 is driven by multiple, interconnected layers, including EBV-associated factors, tumor cell-intrinsic oncogenic signaling pathways, and immune feedback-mediated regulatory mechanisms.

Among these factors, LMP1 represents the most critical viral driver of PD-L1 upregulation. Through its C-terminal activation regions (CTARs), LMP1 recruits adaptor proteins such as tumor necrosis factor receptor-associated factors (TRAFs) and tumor necrosis factor receptor type 1-associated death domain protein (TRADD), thereby constitutively activating the NF-κB and MAPK signaling axes and promoting PD-L1 transcription and expression [13,14]. Studies have shown that LMP1 not only enhances the expression of membrane-bound PD-L1 on tumor cells but is also associated with elevated levels of circulating soluble PD-L1 (sPD-L1) and unfavorable clinical outcomes [13]. In addition, LMP1 induces the secretion of immunosuppressive cytokines, including IL-6 and IL-10 [15]. IL-6 activates the JAK/STAT3 pathway, leading to sustained STAT3 phosphorylation and direct binding of STAT3 to the PD-L1 promoter, thereby enhancing PD-L1 transcription [16]. IL-10, by suppressing antitumor immune responses and promoting the differentiation of Tregs, further contributes to the establishment of an immunosuppressive microenvironment [15,17]. Notably, the IL-6/IL-10–STAT3 axis can, in turn, upregulate LMP1 expression, forming a positive LMP1–STAT3 feedback loop that sustains the persistent coupling of viral and host signaling pathways [18].

Beyond viral factors, tumor cell-intrinsic oncogenic signaling pathways also contribute to the sustained upregulation of PD-L1. Among these, constitutively activated STAT3 can directly bind to the PD-L1 promoter and drive its high-level expression. Pharmacologic or genetic inhibition of STAT3 activity reduces PD-L1 expression and partially restores T-cell function [16], suggesting that therapeutic strategies targeting STAT3 may enhance responsiveness to immune checkpoint blockade. In addition to STAT3-mediated transcriptional activation, recent evidence has identified Early Growth Response 1 (EGR1) as an additional transcriptional regulator of PD-L1 in NKTCL. EGR1 directly binds to the PD-L1 promoter and enhances its transcription, thereby contributing to immune evasion [19].

In addition, NKTCL tumor cells exhibit a characteristic pattern of adaptive immune resistance. Under immune selective pressure—such as interferon-γ (IFN-γ) stimulation or following PD-1 blockade—tumor cells can further upregulate PD-L1 through activation of the JAK/STAT1 pathway, thereby attenuating T-cell-mediated cytotoxicity and promoting either primary or acquired resistance [20,21].

Beyond canonical LMP1-driven signaling, the immunobiological effects of EBV in NKTCL may also be shaped by viral heterogeneity and latency-associated differences in gene expression. EBV latency can be broadly categorized into latency I, II, and III programs, which differ in latent gene-expression patterns. NKTCL is generally considered to be associated predominantly with a latency II program, but the expression of latent viral antigens such as LMP1 may still be heterogeneous or dynamically regulated across biologic contexts, with potential implications for immune evasion and therapeutic targetability [2,22,23]. In addition, EBV-associated immunosuppressive cytokine programs, including viral IL-10-related effects, may further dampen antigen presentation and cytotoxic immune responses, thereby reinforcing the suppressive microenvironment [24]. Moreover, recurrent EBV genomic alterations, including deletions affecting regions such as RPMS1 and OriP, may influence viral persistence, transcriptional regulation, and host immune interaction, although their precise functional and therapeutic implications in NKTCL remain to be fully defined [25,26,27]. Collectively, these features suggest that EBV in NKTCL should be viewed not only as a driver of PD-L1 upregulation, but also as a biologically dynamic determinant of immune escape with potential relevance to the design, selection, and durability of EBV-directed therapeutic strategies [22,25].

Overall, PD-L1 expression in NKTCL is governed by a multilayered regulatory network involving EBV viral proteins, tumor cell-intrinsic oncogenic signaling, immune feedback mechanisms, and viral heterogeneity. This mechanistic framework not only provides a biological rationale for PD-1/PD-L1 blockade in NKTCL but also supports EBV-directed therapeutic approaches beyond checkpoint inhibition, including adoptive cellular therapies and therapeutic vaccination. At the same time, viral antigen heterogeneity and latency-associated expression variability may influence target selection and treatment durability.

### 2.2. Clinical Efficacy and Evidence Summary of PD-1/PD-L1 Inhibitors

Given the profoundly immunosuppressive tumor immune microenvironment driven by EBV–LMP1 signaling in NKTCL, ICIs targeting the PD-1/PD-L1 pathway have progressively emerged as an important therapeutic strategy for this disease. To date, the most extensively reported ICIs include multiple PD-1 inhibitors—such as sintilimab, pembrolizumab, nivolumab, camrelizumab, tislelizumab, and toripalimab—as well as PD-L1 inhibitors, including avelumab and sugemalimab. By blocking PD-1/PD-L1-mediated inhibitory signaling, these agents restore the effector functions of T cells and NK cells and have demonstrated clinically meaningful antitumor activity in patients with relapsed or refractory NKTCL.

To systematically delineate the clinical evidence of PD-1/PD-L1 inhibitors across different disease settings in NKTCL, this review integrates data from prospective phase I–III clinical trials, retrospective studies, and case series. Key aspects of study design, therapeutic regimens, and major efficacy and safety outcomes are summarized in Table 1. Given the differences in disease setting, treatment backbone, prior therapy, sample size, and study maturity, cross-trial comparisons should be interpreted cautiously.

Table 1 summarizes the current clinical evidence of PD-1/PD-L1 inhibitors across distinct clinical settings in NKTCL. In early-stage disease, encouraging activity has been reported with PD-1 blockade-based combination strategies, although much of the evidence remains immature. In advanced disease, promising efficacy signals have mainly emerged from small phase II studies and retrospective analyses of first-line combination regimens, although hematologic toxicity remains a relevant concern. In relapsed or refractory NKTCL, clinical activity appears more heterogeneous, particularly with monotherapy-based approaches, whereas selected combination strategies have shown encouraging response depth in early studies. For NKTCL-associated HLH, immunotherapy-based regimens remain exploratory, with definitive evidence still emerging.

Beyond conventional PD-1-based combinations, exploratory intensification of the immunochemotherapy backbone has been reported in advanced NKTCL. In a small retrospective cohort, P3-GemOx, a liposomal mitoxantrone-intensified anti-PD-1-based GemOx-containing regimen, achieved an ORR of 100%, compared with 63.6% in the PP-GemOx group, with more patients proceeding to HSCT. Although preliminary survival outcomes appeared encouraging, the limited sample size and retrospective design warrant cautious interpretation and prospective validation [28].

Despite these encouraging findings, several challenges remain for the clinical application of ICIs in NKTCL. A subset of patients exhibits suboptimal responses to ICI monotherapy or develops secondary resistance, which may be attributable to persistent activation of EBV–LMP1-associated signaling, increased infiltration of immunosuppressive cellular populations, and the co-expression of multiple immune checkpoint molecules within the tumor immune microenvironment. Although combination regimens can enhance the depth of response, they are frequently accompanied by increased hematologic toxicity and immune-related adverse events. Moreover, clinical evidence supporting PD-L1 inhibitors remains relatively limited, and their optimal timing of use, appropriate patient selection, and strategies for integration with chemotherapy or targeted agents require further validation in prospective clinical studies. However, the marked heterogeneity of clinical responses across different disease settings indicates that PD-1/PD-L1 activation alone does not fully account for therapeutic sensitivity in NKTCL, thereby underscoring the need to examine resistance mechanisms more systematically.

**Table 1:** Major clinical studies of PD-1/PD-L1 inhibitors across different disease settings in NKTCL.

Population	Drug	Target	Clinical Phase	Study Status	N	Combination Regimen	Key Outcomes	Key Adverse Events	NCT No.	Reference
Early-stage NKTCL	Toripalimab	PD-1	Phase III	Recruiting	207 (planned)	Sequential chemoradiotherapy ± toripalimab	NR	NR	NCT04365036	/
	Pembrolizumab	PD-1	Phase II	Active, not recruiting	19	/	NR	NR	NCT03728972	/
	Sintilimab	PD-1	Phase II	Completed	49	Anlotinib + pegaspargase, sandwiched with radiotherapy	ORR 87.8%, CRR 87.8%; 2-year PFS 87.6%; 2-year OS 97.9%	Grade 3–4 TRAEs 41.4%; grade 3–4 hematologic AEs 6.9%; no TRD	NCT03936452	[29]
	Sintilimab	PD-1	Phase II	Unknown status	30 (planned)	Pegaspargase combined with radiotherapy	NR	NR	NCT04676789	/
	Tislelizumab	PD-1	Phase II	Unknown status	54 (planned)	P-GEMOX combined with radiotherapy	NR	NR	NCT05254899	/
	Tislelizumab	PD-1	Phase II	Recruiting	38 (planned)	Radiotherapy	NR	NR	NCT05477264	/
	PD-1 inhibitor	PD-1	Phase II	Unknown status	35 (planned)	Pegaspargase + Chidamide	NR	NR	NCT04414969	/
	Camrelizumab	PD-1	Phase N/A	Unknown status	60 (planned)	Pegaspargase + Apatinib combined with radiotherapy	NR	NR	NCT04366128	/
Advanced NKTCL	Sintilimab	PD-1	Phase II	Active, not recruiting	34	P-GEMOX	ORR 100%; CRR 85%; 24-mo PFS 64%; 36-mo OS 76%	Grade 3–4 TRAEs: 41.4% (hematologic: 6.9%); no TRD	NCT04127227	[30]
	Sintilimab	PD-1	Phase II	Unknown status	22	Pegaspargase	ORR 68%; CRR 59%; 2-yr PFS 68%; 2-yr OS 86%	Grade 3–4 AEs: 45% (neutropenia: 32%); no TRD	NCT04096690	[31]
	Sintilimab	PD-1	Phase II	Recruiting	84 (planned)	P-GEMOX	NR	NR	NCT06583083	/
	Sintilimab	PD-1	Phase II	Unknown status	37 (planned)	Anlotinib + Pegaspargase	NR	NR	NCT04004572	/
	PD-1 inhibitor	PD-1	Retrospective study	/	9	P-GEMOX	ORR 88.9% (7 CR, 1 PR); CRR 77.8%; 1-yr PFS 66.7%; 1-yr OS 100%	Grade 3–4 AEs were observed (anemia, neutropenia, and thrombocytopenia; each 33.3%); no TRD	/	[32]
R/R NKTCL	Sugemalimab	PD-L1	Phase III	Not yet recruiting	150 (planned)	P-GEMOX	NR	NR	NCT05700448	/
	Camrelizumab	PD-1	Phase II	Unknown status	97 (planned)	/	NR	NR	NCT03363555	/
	Camrelizumab	PD-1	Phase II	Unknown status	61 (planned)	Apatinib	NR	NR	NCT03701022	/
	Pembrolizumab	PD-1	Phase II	Unknown status	20 (planned)	/	NR	NR	NCT03107962	/
	Pembrolizumab	PD-1	Phase II	Unknown status	33 (planned)	/	NR	NR	NCT03021057	/
	Sintilimab	PD-1	Phase II	Completed	28	/	ORR 75%; CRR 21.4%; 24-month OS 78.6%	Grade 1–2 TRAEs: 60.7% (lymphocyte count decreased: 42.9%); SAEs: 25.0%; no TRD.	NCT03228836	[33]
	Sintilimab	PD-1	Phase II	Unknown status	20 (planned)	Lenalidomide	NR	NR	NCT04231370	/
	Sintilimab	PD-1	Phase II	Unknown status	20 (planned)	Decitabine	NR	NR	NCT04279379	/
	Tislelizumab	PD-1	Phase II	Completed	22	/	ORR 31.8%; CRR 18.2%; mPFS 2.7 months; mOS 8.8 months	Grade ≥ 3 TRAEs: 31.8%; no TRD	NCT03493451	[34]
	Tislelizumab	PD-1	Phase II	Unknown status	50 (planned)	Dexamethasone + Azacytidine + Pegaspargase	NR	NR	NCT04899414	/
	Tislelizumab	PD-1	Phase II	Unknown status (preliminary results reported)	10	Chidamide + Lenalidomide + Etoposide	ORR 87.5%, CRR 62.5% (preliminary)	Grade ≥ 3 neutropenia: 60%; irAEs: 2 patients (Grade 1 hypothyroidism); no TRD	NCT04038411	[35]
	Avelumab	PD-L1	Phase II	Unknown status (results published)	21	/	ORR 38%; CRR 24%; mPFS 2.7 months; mOS not reached	Most TRAEs were grade 1–2; grade 3 TRAEs mainly neutropenia (10%); no grade 4 TRAEs or TRD.	NCT03439501	[36]
	Avelumab	PD-L1	Phase II	Active, not recruiting	23	/	NR	NR	NCT04414163	/
	Sugemalimab	PD-L1	Phase II	Completed	80	/	ORR 44.9%; CRR 35.9%; 12-month DoR rate 82.5%; mOS not reached	Most TRAEs were grade 1–2; grade 3–4 TRAEs 16.3%; no TRD	NCT03595657	[37]
	Camrelizumab	PD-1	Phase I/II	Recruiting	43 (planned)	Pegaspargase + Linperlisib	NR	NR	NCT06376721	/
	Pembrolizumab	PD-1	Phase I/II	Terminated	17 (terminated)	Copanlisib	NR	NR	NCT02535247	/
	Sintilimab	PD-1	Phase Ib/II	Completed	38	Chidamide	ORR 59.5%; CR 48.6%; mPFS 23.2 months; mOS 32.9 months	Grade ≥ 3 TRAEs mainly neutropenia (28.9%); irAEs 47.4%; no TRD	NCT03820596	[38]
	Tislelizumab	PD-1	Phase N/A	Completed	62	azacytidine, lenalidomide or etoposide, pegaspargase	NR	NR	NCT05058755	/
	Tislelizumab	PD-1	Phase Ib/II	Active, not recruiting	46 (planned)	Mitoxantrone Hydrochloride Liposome	NR	NR	NCT05464433	/
	IBI318	PD-1/PD-L1	Phase Ib/II	Terminated	9 (terminated)	/	NR	NR	NCT04602065	/
	Pembrolizumab	PD-1	Retrospective study	/	59	/	ORR 40.7%; CRR 28.8%; 2-yr PFS 21.5%; 2-yr OS 28.7%	Grade 3–4 AEs 20.3% (neutropenia: 10.2%); no TRD	/	[39]
	Nivolumab	PD-1	Case series	/	3	/	ORR 100% (2 CR, 1 PR); CRR 66.7%	CRS and TLS reported; no typical irAEs	/	[40]
	Pembrolizumab	PD-1	Case series	/	7	/	ORR 100% (5 CR, 2 PR); CRR 71.4%	One case of grade 2 GVHD after allo-HSCT; no other TRAEs	/	[41]
NKTCL-HLH	Sintilimab	PD-1	Phase II	Unknown status	37 (planned)	Chidamide and Azacitidine	NR	NR	NCT05008666	

Note: Cross-trial comparisons should be interpreted cautiously because of differences in disease setting, treatment backbone, prior therapy, sample size, and study maturity. Abbreviations: AE, adverse event; allo-HSCT, allogeneic hematopoietic stem cell transplantation; CR, complete response; CRR, complete response rate; CRS, cytokine release syndrome; DoR, duration of response; GVHD, graft-versus-host disease; HLH, hemophagocytic lymphohistiocytosis; irAE, immune-related adverse event; mOS, median overall survival; mPFS, median progression-free survival; mo, month; N, number of patients; N/A, not available; NKTCL, natural killer/T-cell lymphoma; NR, not reported; ORR, objective response rate; OS, overall survival; PD-1, programmed cell death protein 1; PD-L1, programmed death-ligand 1; PFS, progression-free survival; P-GEMOX, pegaspargase, gemcitabine, and oxaliplatin; PR, partial response; R/R, relapsed or refractory; SAE, serious adverse event; TLS, tumor lysis syndrome; TRAE, treatment-related adverse event; TRD, treatment-related death; yr, year.

### 2.3. Primary and Acquired Resistance to Immunotherapy in NKTCL

#### 2.3.1. Primary Resistance

Although PD-1/PD-L1 inhibitors have demonstrated clinically meaningful efficacy in a subset of patients with relapsed or refractory NKTCL, primary and acquired resistance remain major barriers to durable benefit. Mechanistic insight into resistance pathways will therefore be pivotal for the rational optimization of future immunotherapeutic strategies.

Primary resistance to immunotherapy in NKTCL refers to the failure to achieve an initial meaningful response because of pre-existing tumor-intrinsic and microenvironmental barriers [42,43]. In NKTCL, this is mainly driven by a suppressive EBV-shaped TIME, defective antigen presentation, and baseline co-inhibitory signaling. Although NKTCL lesions often exhibit abundant immune cell infiltration, their composition and functional state are frequently imbalanced and immunosuppressive. Single-cell sequencing analyses have demonstrated a reduced proportion of normal NK cells in tumor lesions compared with peripheral blood, accompanied by enrichment of exhausted T-cell subsets, Tregs, and M2-like macrophages, collectively contributing to a highly suppressive tumor immune microenvironment (TIME) [44]. Spatial transcriptomic studies have further demonstrated a significant accumulation of EBV^+^ macrophages in patients with progressive disease, with their spatial distribution closely associated with poor sensitivity to chemotherapy [45]. Concurrently, an LMP1^+^ malignant NK-cell subset has been shown to secrete dipeptidyl peptidase 4 (DPP4), which cleaves CXCL2, CXCL9, and CXCL10, thereby attenuating the chemotaxis of CXCR2^+^CXCR3^+^ normal NK cells. These cells also exhibit high expression of PD-L1 and CD86, consistent with enrichment of PD-1 signaling and CD86–CTLA-4 interactions, collectively reinforcing a highly immunosuppressive tumor microenvironment [44]. In addition, downregulation of major histocompatibility complex class I (HLA-I) molecules and β2-microglobulin impairs antigen presentation, further weakening T-cell-mediated antigen recognition [46].

An intrinsic network of multiple co-inhibitory receptors on T cells also constitutes a critical component of primary resistance. In NKTCL, T cells frequently co-express PD-1, lymphocyte activation gene-3 (LAG-3), and T-cell immunoglobulin and mucin domain-containing protein-3 (TIM-3), exhibiting a deeply exhausted phenotype. Among these, LAG-3 suppresses TCR signaling through binding to major histocompatibility complex class II (MHC-II) molecules [47,48], whereas interactions between TIM-3 and galectin-9 can induce T-cell apoptosis or functional inactivation [49,50]. Collectively, these pathways form a cooperative inhibitory network that markedly diminishes the cytotoxic capacity of CD8^+^ T cells [51]. Collectively, these findings suggest that blockade of the PD-1/PD-L1 axis alone may be insufficient to fully restore effector T-cell function in NKTCL, potentially contributing to primary resistance.

#### 2.3.2. Acquired Resistance

Acquired resistance to immunotherapy in NKTCL refers to the loss of therapeutic sensitivity after an initial response or disease control under treatment pressure [20,21,46]. It is largely associated with adaptive responses under immune selective pressure. Following immune activation, IFN-γ produced by effector immune cells can drive secondary upregulation of PD-L1 through the JAK1/2–STAT1 axis, thereby establishing a “treatment–feedback” loop that attenuates sustained therapeutic efficacy [20,21]. In addition, tumor cells may undergo metabolic reprogramming through activation of the noncanonical NF-κB pathway, resulting in enhanced aerobic glycolysis and increased lactate production, which may contribute to the establishment of an immunosuppressive tumor microenvironment [52]. Beyond glycolytic rewiring, recent single-cell and spatial transcriptomic analyses have identified fatty acid-binding protein 5 (FABP5) as a metabolic regulator enriched in malignant cells in NKTCL. Its expression correlates with enhanced lipid metabolism and immunosuppressive features, suggesting that FABP5 may represent a potential metabolic vulnerability and therapeutic target in NKTCL [53]. Aberrant angiogenesis and excessive extracellular matrix deposition can further impede T-cell infiltration, thereby constraining the depth and durability of antitumor immune effects [54,55]. In this context, acquired resistance in NKTCL may reflect a progressive loss of therapeutic sensitivity after an initial response, driven by adaptive checkpoint signaling and metabolic remodeling under immune and therapeutic pressure [20,21,52,53]. Emerging single-cell and spatial transcriptomic evidence suggests that treatment-relevant intratumoral heterogeneity and evolving malignant-cell states may also contribute to acquired resistance in NKTCL. However, direct evidence specifically linking clonal development to immunotherapy resistance in this disease remains limited [53].

Collectively, resistance to PD-1/PD-L1 inhibitors in NKTCL is driven by a constellation of mechanisms, including enrichment of immunosuppressive cellular populations, activation of multiple immune checkpoint pathways, defects in antigen presentation, metabolic reprogramming, and the establishment of physical barriers within the tumor microenvironment. Although some resistance mechanisms may overlap between these two categories, distinguishing primary from acquired resistance remains conceptually useful for understanding whether immune evasion is predominantly pre-existing or adaptively reinforced under treatment pressure. Future strategies to overcome resistance will need to shift toward rational, multi-target combination interventions aimed at restoring and sustaining durable antitumor immune responses. The major resistance mechanisms discussed above, together with the corresponding mechanism-based therapeutic strategies and representative therapeutic examples, are summarized in Table 2. These examples include both approaches that have already been explored in NKTCL and mechanism-based potential strategies that remain to be directly evaluated in this disease. Importantly, some of these resistance-associated features may help define biologically relevant stratification states and may also serve as candidate monitoring biomarkers, although prospective validation remains needed. Their potential relevance to treatment stratification and disease monitoring is summarized in Table 3.

### 2.4. Emerging Immune Checkpoints and Multi-Target Immune Blockade Strategies

#### 2.4.1. Emerging Checkpoints (LAG-3/TIM-3)

Beyond the PD-1/PD-L1 pathway, other immune checkpoints, including LAG-3 and TIM-3, also play important roles in immune evasion in NKTCL. As discussed above, their co-expression with PD-1 supports the rationale for therapeutic targeting of these “secondary” immune checkpoints. Accordingly, therapeutic targeting of these “secondary” immune checkpoints is considered a potential strategy to overcome resistance to PD-1 monotherapy and to enhance the efficacy of immunotherapeutic interventions.

At present, inhibitors targeting LAG-3 and TIM-3 have entered early-phase clinical development across a range of solid tumors and lymphomas. For example, the anti-LAG-3 monoclonal antibody Sym022 is being evaluated in a phase I dose-escalation and expansion study as monotherapy in patients with relapsed or refractory solid tumors and lymphomas (with lymphoma subtypes not specifically defined) (NCT03489369), and in combination with Sym021 (anti-PD-1) or Sym023 (anti-TIM-3) as an exploratory treatment for relapsed or refractory solid tumors (NCT04641871). Although clinical trials specifically dedicated to NKTCL are currently lacking, the frequent co-expression of LAG-3 and TIM-3 with PD-1 and the biological rationale for multi-checkpoint blockade have been well supported by pan-cancer studies, providing a strong theoretical foundation for further investigation of these strategies in NKTCL [64,65,66,67].

#### 2.4.2. PD-1-Based Bispecific ICIs (PD-1 × LAG-3/TIM-3/CTLA-4/TIGIT)

Building on the cooperative inhibitory roles of secondary immune checkpoints such as LAG-3 and TIM-3 in NKTCL, bispecific immune checkpoint inhibitors (bispecific ICIs) have emerged as a novel therapeutic strategy. The central advantage of these agents lies in their ability to simultaneously relieve inhibition mediated by PD-1 and another key exhaustion pathway within a single molecular framework, potentially enhancing antitumor T-cell activity [66]. In recent years, early-phase studies of this class of agents across multiple solid tumors and hematologic malignancies have demonstrated favorable safety profiles and preliminary antitumor activity.

Among representative agents, the phase I study of the PD-1 × LAG-3 bispecific antibody tebotelimab (MGD013) (NCT03219268) has demonstrated favorable tolerability and observable antitumor activity across multiple refractory solid tumors and hematologic malignancies, with relapsed/refractory diffuse large B-cell lymphoma (R/R DLBCL) as a representative subtype [68]. Similarly, the first-in-human phase I study of another PD-1 × LAG-3 bispecific antibody, EMB-02, has shown comparable tolerability and early signals of efficacy in patients with advanced solid tumors [69]. Along the TIM-3 axis, the PD-1 × TIM-3 bispecific antibody lomvastomig (RO7121661) is currently being evaluated in a phase I study in patients with advanced solid tumors (NCT03708328). Evidence from other tumor types, particularly melanoma and lung adenocarcinoma models, has suggested that TIM-3 may be upregulated in the setting of resistance to PD-1 blockade, providing a broader biologic rationale for combined blockade of TIM-3 and PD-1, although direct evidence specific to NKTCL remains limited [66]. In addition, the PD-1 × CTLA-4 bispecific antibody cadonilimab (AK104) has established a robust foundation of safety and antitumor activity in phase I/II studies across multiple solid tumors [70] and has further demonstrated encouraging therapeutic signals in EBV-associated nasopharyngeal carcinoma [71], supporting its potential translational relevance in NKTCL, which is likewise driven by EBV. Meanwhile, the PD-1 × TIGIT bispecific antibody rilvegostomig (AZD2936) has also shown acceptable safety and preliminary efficacy in patients with immunotherapy-refractory advanced non-small cell lung cancer (NSCLC) (NCT04995523) [66].

Overall, bispecific ICIs, as an engineered extension of combined immune checkpoint blockade, are currently supported primarily by evidence from solid tumors and a limited number of B-cell lymphomas. Nevertheless, their mechanisms of action closely align with the characteristic features of NKTCL—namely, multi-checkpoint co-expression and profound T-cell exhaustion—thereby highlighting their potential translational relevance in this disease.

## 3. Antibody-Based Targeted Therapies

### 3.1. Mechanistic Basis

Targeted monoclonal antibodies exert their antitumor effects through specific recognition of tumor-associated antigens and rely on IgG1 Fc-mediated effector functions, thereby occupying an important position within the therapeutic landscape of NKTCL. The principal antitumor mechanisms include antibody-dependent cellular cytotoxicity (ADCC) and complement-dependent cytotoxicity (CDC). The efficacy of these mechanisms is determined not only by factors such as target antigen density, antibody conformation, and Fc–Fcγ receptor affinity, but is also profoundly influenced by the immunosuppressive tumor immune microenvironment that is characteristic of NKTCL.

During ADCC, engagement of the antibody Fc domain with Fcγ receptor IIIa (FcγRIIIa, CD16) expressed on effector cells—such as NK cells and macrophages—triggers effector-cell activation and the release of cytotoxic mediators, including perforin and granzymes, resulting in tumor cell lysis or apoptosis. The magnitude of ADCC is influenced by multiple factors, including the affinity between the antibody Fc structure and FcγRIIIa, the number and functional status of effector cells (particularly NK cells), and the level of target antigen expression. Fc-engineered antibodies with afucosylated Fc domains have been shown to markedly enhance FcγRIIIa binding, thereby amplifying ADCC activity [72]. In NKTCL, CD38 is widely expressed and is associated with adverse prognosis, making it a potential target for monoclonal antibody-based therapy [73]. Although CD30 exhibits a degree of expression heterogeneity, it likewise represents a plausible therapeutic target [74,75,76]. Notably, when the target antigen is concomitantly expressed on effector cells, antibody engagement may lead to transient depletion of these cells; for example, daratumumab binding to CD38^+^ NK cells can induce temporary NK-cell depletion, thereby potentially attenuating early ADCC responses [77].

Conversely, CDC is initiated when antibody binding to tumor cells recruits C1q, thereby activating the classical complement cascade and leading to the formation of the membrane attack complex (MAC). Tumor cells can express complement regulatory proteins such as CD55 and CD59 that suppress CDC activity, and even antibodies with strong C1q-binding capacity, such as daratumumab, may thus exhibit limited CDC activity, suggesting that complement evasion can influence therapeutic efficacy [78]. Based on these mechanistic features, multiple targeted antibodies have entered clinical evaluation in NKTCL, with their effectiveness showing marked variability in relation to tumor microenvironmental characteristics.

### 3.2. Representative Targeted Antibodies (CD38/CD30)

In clinical studies of R/R NKTCL, antibodies targeting CD38 and CD30 have demonstrated distinct single-agent efficacies. Daratumumab is, to date, the only anti-CD38 monoclonal antibody that has been evaluated as monotherapy in a prospective clinical trial specifically in NKTCL. In the phase II NKT2001 study (NCT02927925), daratumumab achieved an ORR of 25%, with no complete responses (CR) observed, and a median duration of response of less than two months, indicating limited single-agent activity [79]. Immunologic analyses suggest that the efficacy of daratumumab in NKTCL may be influenced by multiple immunologic features, including treatment-associated depletion of NK cells, alterations in T-cell subsets, enrichment of suppressive myeloid populations, and expression of complement inhibitory proteins such as CD55 and CD59. Collectively, these factors may modulate the ADCC- and CDC-mediated antitumor effects of daratumumab [80,81]. In addition, a reported case study suggested that sequential daratumumab followed by PD-1 blockade may be counterproductive in NKTCL, potentially because residual anti-CD38 activity may deplete PD-1-reinvigorated CD38-positive effector T cells, indicating that treatment sequencing may critically influence clinical outcome [82]. Isatuximab, another anti-CD38 monoclonal antibody, possesses multiple mechanisms of action, including ADCC, CDC, and direct induction of tumor cell death. Mechanistically, the spectrum of effector functions triggered by isatuximab appears to be influenced by CD38 density. However, to date, no prospective single-agent clinical data specific to NKTCL have been reported, and its potential role remains primarily inferred from mechanistic considerations and evidence extrapolated from other tumor types [83]. More recently, however, a phase II study of isatuximab plus cemiplimab in relapsed/refractory ENKTL reported encouraging and durable activity, with a best complete response rate of 51% and an objective response rate of 65% [84]. Together, these observations suggest that the therapeutic effects of CD38-targeted strategies in NKTCL may depend on the specific antibody platform, treatment schedule or sequencing, and broader disease context.

In contrast, the CD30-targeted antibody–drug conjugate (ADC) brentuximab vedotin (BV) has demonstrated more consistent single-agent activity in NKTCL. In a phase II study enrolling patients with EBV^+^ lymphomas exhibiting CD30 expression ≥ 1% (including NKTCL), BV monotherapy achieved an ORR of 48% and a CR rate of 20%, with a median duration of response (DoR) approaching 10 months; notably, therapeutic efficacy did not show a strict linear correlation with the level of CD30 expression [85]. In another phase II study of high-CD30-expressing non-Hodgkin lymphomas, BV monotherapy showed activity in the T/NK-cell lymphoma subgroup, including NKTCL. The NKTCL subset (n = 7) achieved an ORR of 28.6% (1 CR and 1 PR). Although the sample size was small and subgroup-specific durability data were not reported, these results support the biological feasibility of CD30-targeted therapy in T/NK-cell-derived tumors [86]. In addition, case-based evidence has described successful treatment of refractory NKTCL with BV, including complete remission achieved in a CD30-positive case report [87]. A subsequent systematic review further noted that some responding cases exhibited relatively low CD30-positive rates (approximately 30%), highlighting that the optimal CD30 cutoff for BV therapy in NKTCL remains uncertain [76]. Taken together, current evidence indicates differential single-agent activity between CD30-targeted ADC therapy and anti-CD38 monoclonal antibodies in NKTCL; however, direct comparative data are lacking.

### 3.3. Other Targets

Beyond the clinically validated targets CD38 and CD30, CD70 has emerged as a target of interest owing to its aberrant upregulation in EBV-associated lymphomas, including NKTCL, where CD70 expression correlates with LMP1-driven oncogenic signaling [88]. Early clinical efforts have explored CD70-based ADCs. MDX-1203 (BMS-936561) was the first CD70-ADC to enter clinical evaluation; in a phase I study (NCT00944905) enrolling 26 patients with relapsed/refractory B-cell non-Hodgkin lymphoma (B-NHL) or renal cell carcinoma (RCC), 69% of patients achieved stable disease (SD), but no partial responses (PR) or CR were observed, indicating limited single-agent antitumor activity [89]. Another CD70-targeted ADC incorporating a pyrrolobenzodiazepine (PBD) dimer payload (SGN-CD70A) was evaluated in a phase I trial (NCT02216890), in which 1 CR and 3 PRs were observed among 20 patients with CD70^+^ NHL; however, myelosuppression, particularly thrombocytopenia, represented the major dose-limiting toxicity [90]. Although clinical efficacy has thus far been modest, these studies provide preliminary evidence supporting the safety and feasibility of CD70-targeted strategies, underscoring CD70 as an important target warranting further optimization and investigation.

In contrast, other molecules such as B7-H3, CCR4, CD52, and CD25, although demonstrating a degree of targetability in pan-cancer studies, remain supported by markedly limited evidence in NKTCL. The targetability of B7-H3 is derived primarily from preclinical data [91]. Antibodies targeting CCR4 and CD52 have shown objective response rates in peripheral T-cell lymphoma (PTCL) and cutaneous T-cell lymphoma (CTCL); however, expression profiling and clinical validation in NKTCL are lacking [92,93]. Exploration of CD25 as a therapeutic target has been initiated in a phase II trial evaluating basiliximab in combination with pegaspargase (NCT04337593), but no results have yet been reported. Overall, with the exception of CD70, these candidate targets in NKTCL remain at the stage of theoretical inference or early exploration, and their clinical value will require confirmation through more systematic molecular characterization and prospective clinical studies.

## 4. Applications and Perspectives of Cellular Immunotherapy in NKTCL

### 4.1. CAR-T Cell Therapy

#### 4.1.1. Preclinical Progress and Emerging Targets

CAR-T therapy—particularly CD19-targeted CAR-T cells—has achieved breakthrough success in B-cell malignancies [94,95,96]. This success largely depends on the stable and high-density expression of lineage-specific antigens such as CD19, as well as a host immune environment that is relatively permissive for effector T-cell expansion and long-term persistence [97].

In contrast, investigation of CAR-T therapy in NKTCL remains at an early exploratory stage, with existing studies largely confined to in vitro experiments and animal models [98]. CAR-T constructs targeting CD38 and EBV–LMP1 have demonstrated robust cytotoxicity across several NKTCL cell lines and achieved effective tumor control in xenograft mouse models; notably, dual-target CD38 × LMP1 CAR-T cells have shown superior capacity to overcome antigen heterogeneity [99]. In parallel, B7-H3—commonly expressed in nasal-type NKTCL—has been validated as a promising CAR target, with corresponding constructs exhibiting potent antitumor activity both in vitro and in vivo, thereby providing a solid foundation for further clinical development [91].

#### 4.1.2. Structural Barriers to Clinical Translation

However, unlike B-cell malignancies, in which CAR-T therapy has achieved durable clinical success through relatively stable lineage-restricted targets, the clinical translation of CAR-T therapy in NKTCL faces multiple structural and biological barriers, particularly target heterogeneity, overlap with normal T/NK-cell antigens, and a profoundly suppressive EBV-driven tumor immune microenvironment. On the one hand, at the level of target antigens, NKTCL lacks an ideal lineage-specific antigen analogous to CD19. Currently explored targets—including CD38, CD56, CD7, and CD30—exhibit substantial heterogeneity in expression across patients and disease stages, and several of these antigens are also expressed on normal T and/or NK cells, thereby increasing the risks of on-target/off-tumor toxicity and CAR-T fratricide [99,100,101]. Although EBV–LMP1, as a virus-associated antigen, offers a degree of tumor specificity, its expression is not constitutive and can be dynamically regulated under host immune pressure and microenvironmental influences, and latency-associated variation in EBV gene expression. This reflects the phenomenon of immune editing in EBV-associated malignancies and may limit the durability of target engagement by LMP1-directed CAR-T therapies [22,102,103]. This variability is broadly consistent with the dynamic biology of EBV latency and may further complicate the durability of LMP1-directed targeting [22,23]. These major candidate antigens, together with their expression heterogeneity and major translational limitations, are summarized in Table 4. On the other hand, the profoundly immunosuppressive TIME driven by EBV constitutes a major constraint on CAR-T cell function. In NKTCL, LMP1-mediated NF-κB activation and oncogenic JAK/STAT3 signaling have been implicated in aberrant PD-L1 upregulation, which can directly dampen effector T-cell activity [13,16,104]. Concurrently, enrichment of Tregs, M2-polarized tumor-associated macrophages, and myeloid-derived suppressor cells collectively establishes an immune ecosystem that is unfavorable for effector T-cell expansion and long-term persistence [44]. In addition, metabolic abnormalities—including lactate accumulation, glucose deprivation, and hypoxia—further suppress CAR-T cell cytotoxicity, rendering it difficult to recapitulate the robust in vivo expansion advantages observed in B-cell malignancies [97].

Moreover, the intrinsic immune status of patients with NKTCL is itself unfavorable for the generation and maintenance of autologous CAR-T cells. Chronic EBV infection and prior exposure to multiple lines of chemotherapy frequently lead to profound exhaustion and functional impairment of peripheral T cells, thereby compromising the initial quality of CAR-T products, their in vivo expansion capacity, and long-term persistence. Previous studies have demonstrated that both the quantity and functional state of the starting T-cell population are among the key determinants of durable CAR-T efficacy, a challenge that is particularly pronounced in non-B-cell-derived malignancies [97]. Collectively, these factors help to explain why multiple CAR-T constructs have shown measurable activity in preclinical models, yet have proven difficult to translate into substantial clinical benefit [105].

At present, clinical evidence supporting CAR-T therapy in NKTCL remains limited. Existing CAR-T studies targeting CD7 or CD30 (NCT04004637, NCT03049449, NCT04008394) have primarily been conducted in populations with T-cell lymphomas or CD30^+^ lymphomas; although NKTCL may be included within the eligibility criteria, no subtype-specific efficacy data for NKTCL have been reported to date. More NKTCL-focused approaches, such as anti-CD56 CAR-T therapy (NCT05941156), have entered phase I clinical evaluation; however, efficacy outcomes have not yet been disclosed.

Overall, the clinical translation of CAR-T therapy in NKTCL remains constrained by the lack of ideal lineage-specific target antigens, the EBV-driven immunosuppressive tumor immune microenvironment, and impaired host T-cell function.

**Table 4:** Expression Heterogeneity and Therapeutic Implications of Key CAR-T Target Antigens in NKTCL.

Target Antigen	Core Expression Features	Clinical Correlations	Key Advantages	Major Limitations	Reference
CD38	Expressed in tumor cells but highly heterogeneous; also present on normal immune cells.	Clinical evidence for CD38-CAR-T in NKTCL remains very limited and is largely extrapolative or conceptual.	Clear biological rationale and good translational feasibility.	Heterogeneous expression may reduce efficacy and promote antigen escape; on-target/off-tumor toxicity is a concern.	[80,99,100,105]
CD56	A classic marker of NKTCL, but expression is variable; normal NK cells also express CD56.	An early CD56 CAR-T study has been registered (NCT05941156), but efficacy results are unavailable.	Highly consistent with the NKTCL phenotype.	Variable expression may increase antigen escape risk; off-tumor effects on normal NK cells are possible.	[100,105,106]
CD7	A pan-T/NK antigen expressed on normal T cells and some NK cells; tumor expression may also be heterogeneous.	Relevant CAR-T studies are mainly basket trials (NCT04004637); NKTCL is an eligible subtype, but efficacy results are unavailable.	Broad target coverage and substantial platform experience.	Limited tumor specificity; fratricide, T-cell depletion, and antigen loss may affect durability.	[100,105,106]
CD30	Expressed in subsets of NKTCL with variable positivity; normal tissue expression is generally low.	Most data come from basket trials (NCT03049449, NCT04008394); NKTCL-specific efficacy data are lacking.	More suitable for CD30-positive subgroups, with theoretically lower off-tumor risk.	Restricted to CD30-positive cases and affected by expression variability; patient selection is required.	[100,105,107]
EBV-LMP1	A virus-associated antigen with relative tumor specificity, but expression is dynamic.	Theoretically more specific, but dynamic expression may limit durable targeting; clinical evidence remains insufficient.	May reduce systemic off-tumor risk and fits EBV-directed targeting logic.	Dynamic expression and immune editing may promote escape; multi-antigen or combination strategies may be needed.	[22,23,99,102,103,105]

Abbreviations: CAR-T, chimeric antigen receptor T-cell; NKTCL, natural killer/T-cell lymphoma; NK, natural killer; EBV, Epstein–Barr virus; LMP1, latent membrane protein 1.

#### 4.1.3. Strategies to Overcome Translational Barriers and Future Engineering Directions

In light of these multiple structural constraints, recent CAR-T research in NKTCL has gradually shifted from incremental modifications of conventional CAR designs toward a more comprehensive strategic realignment centered on disease-specific biology. Unlike the established paradigm in B-cell malignancies, which relies on stable lineage-restricted antigens, effective cellular therapy for NKTCL requires simultaneous optimization across multiple dimensions—including target selection, adaptation to the immunosuppressive tumor immune microenvironment, and the choice of effector cell sources—rather than merely amplifying CAR activation signals.

At the level of target recognition, research efforts have increasingly shifted from the pursuit of a single ideal antigen toward combinatorial recognition and conditional activation strategies. Logic-gated designs exemplified by synNotch CARs enable effector cells to become activated and execute cytotoxic functions only upon the simultaneous recognition of predefined antigen combinations, thereby enhancing tumor selectivity and reducing off-tumor toxicity in settings characterized by pronounced antigen heterogeneity or overlapping antigen expression between tumor and normal tissues [108]. Recent systematic studies have further highlighted that multi-receptor combinatorial and logic-gating strategies represent a critical engineering direction for addressing the fundamental challenge of the “absence of truly tumor-specific antigens,” particularly in tumor types with substantial antigen heterogeneity or high risk of off-target effects [109].

Concurrently, in response to the profoundly immunosuppressive tumor microenvironment characteristic of EBV-driven NKTCL, CAR-T optimization strategies increasingly incorporate engineered reprogramming of inhibitory signaling pathways rather than relying solely on enhanced activation signaling. Approaches such as cytokine armoring and inhibitory-signal rewiring modules—including PD-1 pathway modulation—enable effector cells to maintain functional activity within suppressive tumor microenvironments and are considered key strategies for overcoming tumor-induced immune suppression [110]. In clinical exploration, CAR-T cells expressing a PD-1–CD28 switch receptor have demonstrated improved expansion capacity and preliminary antitumor activity in patients with PD-L1-high B-cell lymphomas, providing direct evidence supporting the translational potential of this strategy [111].

A more NKTCL-specific direction lies in redefining target selection logic based on the etiologic features of the disease. Given that NKTCL is a prototypical EBV-driven lymphoma, incorporating viral latent antigens or virus-associated pathways into CAR design (such as the aforementioned dual-target CD38 × LMP1 CAR-T) may enhance tumor selectivity and reduce off-tumor risk in the absence of ideal lineage-specific antigens. In parallel, adoptive cellular immunotherapy strategies targeting EBV latent antigens—grounded in this etiologic hallmark—have been shown to be feasible, providing an important conceptual framework for improving tumor selectivity when ideal lineage-restricted targets are lacking (see Section 4.2 below). Accordingly, rather than solely attempting to overcome EBV-mediated immunosuppression, proactively converting viral antigens into therapeutic advantages may be more conducive to establishing differentiated treatment paradigms that balance specificity and safety.

In addition, in response to autologous T-cell exhaustion and limitations in manufacturing accessibility, a structural shift in effector cell platforms is emerging as an important developmental direction. Alternative platforms, including CAR-NKT cells and γδ T cells, are being actively explored as potential complements to conventional CAR-T therapy (see Section 4.3 below).

Overall, the future development of CAR-based cellular therapies in NKTCL is more likely to depend on the integrated design of optimized target recognition, reprogramming of inhibitory signaling pathways, and strategic shifts in effector cell platforms. Such a systematic reconfiguration grounded in disease-specific biological features may be more effective than incremental modifications of individual CAR constructs in facilitating meaningful clinical translation of cellular immunotherapy in this highly aggressive lymphoma subtype.

### 4.2. EBV-CTL Therapy

EBV-CTL therapy involves the ex vivo expansion of cytotoxic T lymphocytes capable of recognizing EBV latent antigens, including LMP1, LMP2A, and EBNA1; upon reinfusion, these cells can eliminate EBV-infected cells, thereby exerting dual antiviral and antitumor effects. Compared with engineered platforms such as CAR-T cells, EBV-CTLs rely on endogenous antigen recognition mechanisms, enabling coverage of multiple viral epitopes, reducing the risk of antigen escape, and generally exhibiting a favorable safety profile. Because EBV latent antigen expression may vary across disease contexts, the multi-epitope recognition profile of EBV-CTLs may provide a potential advantage over approaches that rely on a single viral antigen [22,112]. However, their broader clinical application remains constrained by HLA restriction, manufacturing complexity, and potentially limited durability in heavily pretreated or advanced disease [113,114]. Despite these limitations, EBV-CTL therapy may retain particular value in selected clinical contexts, especially for patients with post-transplant relapse or substantial comorbidities, owing to its favorable tolerability and multi-epitope targeting profile [112,114,115,116].

Early studies have established the safety and biological activity of EBV-CTL therapy. Rooney et al. were the first to apply EBV-CTLs in patients with post-transplant lymphoproliferative disease (PTLD) following allogeneic hematopoietic stem cell transplantation, achieving effective control of viral load without inducing graft-versus-host disease (GVHD) or severe adverse events [116]. Subsequent long-term follow-up demonstrated that infused gene-marked CTLs could persist in vivo for up to 9 years and retain functional activity, supporting the establishment of durable immune surveillance [115]. In EBV-associated lymphomas, additional studies have shown that EBV-CTL therapy can induce sustained remissions, with some patients maintaining complete responses for longer than one year, while exhibiting favorable tolerability [112].

To enhance clinical accessibility, third-party “off-the-shelf” EBV-CTL platforms have been progressively developed. Vickers and colleagues established a GMP-compliant third-party EBV-specific CTL bank covering multiple human leukocyte antigen (HLA) types and reported CR in 8 of 10 patients with PTLD who received infusions, supporting the feasibility and safety of this off-the-shelf approach [113]. Prospective investigations specifically in NKTCL have also yielded encouraging signals: in a multicenter phase II study of 15 patients with advanced NKTCL, autologous EBV-CTL therapy achieved an ORR of 50% and a CR rate of 30% in patients with measurable relapsed/refractory disease, with a median PFS of 3.9 months. The therapy was well tolerated, without cytokine release syndrome (CRS), immune effector cell-associated neurotoxicity syndrome (ICANS), or GVHD [114].

Collectively, EBV-CTL therapy represents one of the most disease-specific cellular immunotherapeutic strategies in NKTCL, with a well-established safety profile and encouraging early efficacy signals. With continued advances in third-party cell bank development, manufacturing standardization, and combination strategies with immune checkpoint inhibitors or epigenetic modulators, the therapeutic potential of EBV-CTLs within the treatment landscape of NKTCL warrants further investigation and holds considerable promise.

### 4.3. Other Emerging Engineered Cellular Therapy Platforms

NKT cells possess both innate and adaptive immune features. Their semi-invariant TCR enables recognition of lipid antigens presented by CD1d molecules, while receptors such as NKG2D and NKp30 allow sensing of tumor-associated stress signals. These properties support tumor recognition even in settings characterized by antigen heterogeneity or impaired antigen presentation [117,118,119]. In addition, the minimal risk of GVHD, intrinsic tumor-homing capacity, and the absence of severe CRS or ICANS reported in early-phase clinical studies support NKT cells as an engineered cellular platform with a favorable safety profile [120,121]. Based on these attributes, CAR-NKT cells have entered early clinical exploration across multiple tumor types and have shown manageable safety profiles with preliminary antitumor activity. Representative examples include GD2-CAR-NKT cells in neuroblastoma [122], allogeneic “off-the-shelf” CD19-CAR-iNKT platforms (NCT03774654, NCT05487651), and CD70-CAR-NKT cells in solid tumors (NCT06182735). However, no direct in vivo models or clinical studies of CAR-NKT therapy specifically targeting NKTCL have yet been reported. Challenges remain with respect to cell sourcing, ex vivo expansion efficiency, and maintenance of effector function within immunosuppressive tumor microenvironments, and relevant engineering optimization strategies remain under active investigation [121].

γδ T cells mediate antitumor effects through MHC-independent mechanisms, including non-classical recognition of phosphoantigens and sensing of tumor-associated stress signals via receptors such as NKG2D. These properties enable γδ T cells to maintain cytotoxic activity in tumor contexts characterized by impaired antigen presentation or antigen heterogeneity [123,124,125]. In addition, their relatively low risk of GVHD confers potential advantages for allogeneic “off-the-shelf” therapeutic platforms [123,124]. Although early clinical studies have demonstrated the overall feasibility and safety of γδ T-cell-based therapies in multiple myeloma, acute myeloid leukemia, and selected solid tumors, their clinical efficacy has been heterogeneous, and in vivo persistence remains limited [123,126]. Given the microenvironmental features of NKTCL—including chronic inflammatory activation, immune evasion, and restricted antigen presentation [1,62]—γδ T cells may, from an immunological standpoint, possess theoretical suitability for this disease. However, to date, direct in vivo models or clinical evidence specifically addressing γδ T-cell therapy in NKTCL are lacking, and their true therapeutic efficacy and in vivo durability await further systematic validation.

## 5. Immune Engagers in Tumor Immunotherapy and Their Potential Application in NKTCL

### 5.1. T-Cell Engagers (TCEs)

In NKTCL, TCEs are of potential interest because tumor-associated antigens such as CD38 and CD70 may provide candidate targets for T-cell redirection. TCEs function by simultaneously binding CD3 on T cells and tumor-associated antigens on malignant cells, thereby rapidly recruiting peripheral T cells to the tumor cell surface and inducing perforin- and granzyme-mediated cytotoxic killing. This mechanism enables direct T-cell activation independently of antigen presentation. In contrast to NK cell engagers, TCEs leverage the abundance and potent cytotoxic capacity of T cells and therefore generally exhibit stronger antitumor killing potential.

In recent years, multiple bispecific antibodies targeting CD3 and tumor-associated antigens have demonstrated preliminary efficacy in hematologic malignancies such as lymphoma and multiple myeloma. For example, the CD38-targeted bispecific antibody ISB1342 (CD38 × CD3) is currently being evaluated in a phase I dose-escalation study in patients with relapsed/refractory multiple myeloma; preclinical data indicate that this agent can induce T cell-mediated cytotoxicity even against cells with low CD38 expression, with a manageable toxicity profile [127]. In addition, another CD38 × CD3 bispecific antibody, XmAb18968, has entered phase I clinical evaluation (NCT05038644) in patients with relapsed/refractory acute myeloid leukemia (AML) and T-cell acute lymphoblastic leukemia (T-ALL), although only conference abstracts describing the trial design have been reported to date [128]. T-cell engagers targeting CD70 are also under active development. CD70 × CD3 bispecific antibodies have demonstrated effective redirection of T cells against CD70^+^ tumor cells in vitro and in xenograft models. However, to date, published early-phase clinical efficacy data in humans remain limited [129]. Although no TCEs have been specifically evaluated in dedicated NKTCL trials to date, their application in this disease is biologically plausible but still awaits clinical validation.

### 5.2. NK Cell Engagers (NKCEs)

In NKTCL, NKCEs are of potential interest as an emerging strategy for redirecting NK-cell cytotoxicity in an EBV-driven and immunosuppressive disease context. In recent years, NKCEs based on bispecific or multispecific antibody architectures have emerged as an important technological direction in tumor immunotherapy. These molecules simultaneously bind activating receptors on the surface of NK cells—such as CD16A, NKG2D, and NKp46—and tumor-associated antigens, including CD19, CD33, CD38, and CD30, thereby redirecting NK cells to mediate targeted tumor cell killing. NKCEs offer several advantages, including “off-the-shelf” availability, low immunogenicity, and a favorable safety profile. Representative formats include bispecific killer cell engagers (BiKEs) and trispecific killer cell engagers (TriKEs), the latter of which incorporate an IL-15 moiety to further enhance NK-cell activation, proliferation, and in vivo persistence.

Early studies demonstrated that CD16A-mediated BiKEs and TriKEs can effectively activate NK cells, promoting degranulation and cytotoxic effector functions, thereby establishing the immunological foundation for NK cell engagers [130]. Subsequently, the BiKE platform was extended to myeloid targets with the development of a CD16 × CD33 BiKE, which recapitulated CD16-mediated direct NK-cell activation in AML and myeloid models and markedly enhanced NK-cell degranulation, cytokine production, and cytotoxicity against CD33^+^ tumor cells [131]. Further refinement led to IL-15-containing TriKEs (161533TriKE), which further augmented NK-cell persistence and functional activity [132]. A representative agent, GTB-3550 (161533TriKE; CD16/IL-15/CD33), showed an acceptable safety profile and induced NK-cell proliferation and activation in a phase I study in patients with relapsed/refractory AML and myelodysplastic syndromes (MDS) (NCT03214666) [133].

Building on the NKCE platform, AFM13 is a tetravalent bispecific antibody targeting CD30 and CD16A that bridges NK cells with CD30^+^ tumor cells and induces ADCC. Its phase I monotherapy study demonstrated favorable safety and controllable immune activation in patients with relapsed/refractory Hodgkin lymphoma (HL) [134]. Preclinical studies further showed that precomplexing AFM13 with memory-like NK cells markedly enhanced cytotoxicity against CD30^+^ tumors and increased IFN-γ secretion [135]. Subsequently, a phase I clinical trial (NCT04074746) evaluating AFM13-precomplexed, cytokine-preactivated cord blood-derived NK cells in patients with relapsed/refractory CD30^+^ lymphomas reported an ORR of 92.9% and a CR of 66.7%. Notably, no CRS, ICANS, or GVHD was observed [136]. Overall, although NKCE-based platforms have shown encouraging activity in other hematologic malignancies, their application in NKTCL remains exploratory, and direct NKTCL-specific clinical evidence is still lacking.

## 6. Targeting Epstein–Barr Virus Vaccines

### 6.1. Prophylactic EBV Vaccines

NKTCL is a prototypical EBV-driven lymphoma, which provides an etiologic rationale for considering EBV vaccine strategies. Early efforts in prophylactic EBV vaccine development focused predominantly on the gp350 antigen. A landmark phase II clinical trial demonstrated that a recombinant gp350 vaccine could reduce the incidence of infectious mononucleosis but failed to completely prevent primary EBV infection, highlighting the intrinsic limitations of a single neutralizing target [137]. Accordingly, subsequent efforts have shifted toward multivalent and multi-antigen vaccine platforms. Structure-guided gH/gL nanoparticle vaccines have shown broader and more potent neutralizing antibody responses in preclinical studies than gp350-based approaches [138]. In addition, newer candidates, including the nanoparticle-based gp350–ferritin vaccine (NCT04645147) and the multivalent glycoprotein mRNA vaccine mRNA-1189 (NCT05164094), are currently undergoing phase I evaluation for safety and immunogenicity [139]. However, in the context of established EBV-associated malignancies such as NKTCL, the direct translational relevance of prophylactic vaccination remains limited, thereby shifting greater attention toward therapeutic vaccine strategies.

### 6.2. Therapeutic EBV Vaccines

In contrast to prophylactic vaccines, which aim to prevent primary infection, therapeutic EBV vaccines are designed to enhance cellular immune responses against latent antigens such as EBV nuclear antigen 1 (EBNA1) and LMP2, thereby overcoming the immunosuppressive state characteristic of EBV-associated malignancies. Peptide- or viral vector-based vaccines targeting EBNA1 and/or LMP2 have been shown in preclinical studies to elicit robust EBV-specific CD8^+^ T-cell responses, establishing an immunological foundation for the treatment of EBV-positive lymphomas [140,141]. This strategy is of particular potential relevance for NKTCL, in which profound immune exhaustion and a highly immunosuppressive tumor microenvironment represent defining biological features.

At present, therapeutic EBV vaccines have begun to enter early-phase clinical exploration in NKTCL, although the overall body of evidence remains limited. Recently, an EBV mRNA-based therapeutic vaccine delivered via lipid nanoparticles (WGc-043) has entered a phase I clinical study in patients with EBV-positive relapsed or refractory lymphomas, with inclusion of NKTCL (ChiCTR2500108428). In parallel, a dedicated mechanistic study of this EBV mRNA vaccine has been initiated (NCT06788600), with a focus on delineating EBV-specific T-cell responses and associated biomarker dynamics, thereby providing immunological support for subsequent efficacy-oriented investigations. In addition, an early-phase basket study is evaluating the same mRNA immunotherapy platform (WGc-043) across EBV-related diseases, with planned use as monotherapy and/or in combination with immune checkpoint inhibitors (NCT07349836).

Although no therapeutic EBV vaccines have yet been specifically designed for NKTCL, and existing studies are limited by small sample sizes, the absence of control arms, and a lack of long-term follow-up data, these exploratory studies provide early clinical signals supporting the feasibility of vaccine-based activation of EBV-specific immunity in NKTCL. However, the therapeutic effectiveness of vaccine-based strategies may also depend on the stability, immunogenicity, and disease-context-specific expression of the targeted EBV latent antigens [22,102]. Looking ahead, combination strategies integrating therapeutic EBV vaccines with PD-1/PD-L1 inhibitors, EBV-specific cellular therapies, and engineered cellular immunotherapeutic platforms may represent an important avenue for reshaping the immune microenvironment of EBV-driven NKTCL.

## 7. Multimodal Immunotherapeutic Combination Strategies

### 7.1. ICIs Combined with Radiotherapy (Radioimmunotherapy)

NKTCL is highly radiosensitive, particularly in patients with localized disease, in whom radiotherapy serves as a therapeutic cornerstone that markedly improves local control and confers long-term survival benefits, thereby providing a strong clinical rationale for combining radiotherapy with ICIs [2,142]. Beyond its direct cytotoxic effects, radiotherapy can induce immunogenic cell death (ICD), leading to the release of tumor antigens and damage-associated molecular patterns (DAMPs), such as high-mobility group box 1 (HMGB1), adenosine triphosphate (ATP), and calreticulin (CRT). These signals promote dendritic cell antigen uptake and cross-presentation, thereby initiating tumor-specific T-cell responses [143,144]. Concurrently, radiotherapy-induced accumulation of cytosolic DNA activates the cyclic GMP–AMP synthase-stimulator of interferon genes (cGAS–STING) pathway and triggers type I interferon signaling, which further enhances the expression of antigen presentation-related molecules and facilitates effector T-cell recruitment. Through these mechanisms, local radiotherapy acquires the potential to function as an “in situ vaccination” modality that primes antitumor immune responses [145].

**Figure 2:**
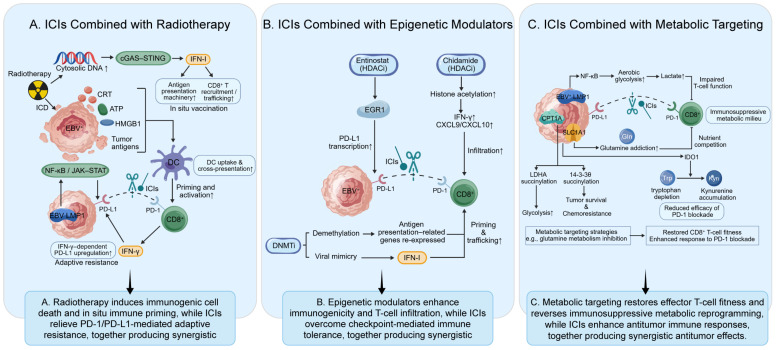
Synergistic Mechanisms of Multimodal Immunotherapeutic Combination Strategies. These combination strategies may exert complementary antitumor effects by enhancing immune priming, increasing tumor immunogenicity, and alleviating immunometabolic suppression. Created with BioGDP.com [12]. Arrows indicate activation, signaling pathways, or mechanistic flow. Abbreviations: ATP, adenosine triphosphate; CRT, calreticulin; DC, dendritic cell; DNMTi, DNA methyltransferase inhibitor; EBV, Epstein–Barr virus; EGR1, early growth response 1; Gln, glutamine; HDACi, histone deacetylase inhibitor; HMGB1, high-mobility group box 1; ICD, immunogenic cell death; ICIs, immune checkpoint inhibitors; IDO1, indoleamine 2,3-dioxygenase 1; IFN-I, type I interferon; IFN-γ, interferon-γ; Kyn, kynurenine; LMP1, latent membrane protein 1; PD-1, programmed cell death protein 1; PD-L1, programmed death-ligand 1; Trp, tryptophan.

However, radiotherapy-induced immune activation is frequently accompanied by IFN-γ-dependent upregulation of PD-L1, resulting in adaptive immune resistance that constrains antitumor T-cell effector function. Blockade of the PD-1/PD-L1 axis can relieve this negative feedback, thereby providing a clear mechanistic basis for synergy with radiotherapy [146,147]. In EBV-driven NKTCL, this synergy is further reinforced by additional biological considerations: EBV latent proteins, particularly LMP1, sustain PD-L1 overexpression through activation of the NF-κB and JAK–STAT signaling axes [13,104,148]. These observations support a strong biological rationale for combining PD-1/PD-L1 inhibitors with radiotherapy to counteract immunosuppression in the context of radiotherapy-induced immune priming. More broadly, the major synergistic mechanisms underlying multimodal immunotherapeutic combination strategies are illustrated in Figure 2.

At present, multiple clinical studies have explored the combination of radiotherapy with PD-1/PD-L1 inhibitors in the treatment of NKTCL. Among these, the CLCG-NKT-2101 study (NCT05149170) is a multicenter, single-arm phase II trial that adopts a risk-adapted strategy to evaluate tislelizumab as induction therapy administered concurrently with radiotherapy in patients with low-risk, early-stage NKTCL. To date, only the study protocol has been published, and no peer-reviewed efficacy or survival data have been reported [149]. In addition, NCT05477264 is investigating concurrent tislelizumab and radiotherapy as first-line treatment for newly diagnosed, localized NKTCL, while NCT04417166 evaluates the efficacy and safety of pembrolizumab combined with involved-field radiotherapy in treatment-naïve NK/T-cell lymphoma patients. In parallel, strategies integrating PD-1 inhibitors with chemotherapy followed by sequential radiotherapy are also being explored. A single-arm, open-label phase II study assessed tislelizumab in combination with gemcitabine, pegaspargase, and etoposide (PPGE), followed by sequential radiotherapy, in patients with localized NK/T-cell lymphoma (ChiCTR2400091671). Preliminary results from this study were presented as an e-Poster at the EHA 2024 Congress, demonstrating encouraging efficacy signals and manageable safety in early-stage NKTCL. Compared with conventional intensified regimens such as SMILE, this approach was associated with lower rates of severe hematologic and non-hematologic toxicities, suggesting that PPGE followed by radiotherapy may represent a more tolerable treatment option [150]. Overall, the current body of evidence primarily supports the feasibility and biological rationale of radioimmunotherapy combinations in NKTCL. Whether these strategies confer an independent efficacy advantage over standard radiotherapy alone remains to be determined and will require confirmation through randomized controlled trials or high-quality comparative cohort studies.

### 7.2. ICIs Combined with Epigenetic Modulators

In NKTCL, profound T-cell exhaustion and impaired antigen presentation limit the therapeutic efficacy of PD-1/PD-L1 blockade. Epigenetic modulators can enhance tumor immunogenicity, amplify interferon signaling, and promote effector T-cell infiltration, thereby augmenting antitumor immune responses and improving the clinical activity of immune checkpoint inhibitors.

Histone deacetylase inhibitors (HDACi) represent one of the most extensively investigated classes of epigenetic modulators. Preclinical studies have demonstrated that chidamide enhances histone acetylation, upregulates CXCL9/10 expression, and activates IFN-γ signaling, thereby exerting synergistic effects when combined with PD-1 blockade. In contrast, the combination of anti-PD1 and romidepsin did not reproduce comparable enhancement of IFN-γ response or T-cell chemokine induction in similar NKTCL immunocompetent models, possibly reflecting structural and HDAC inhibitory differences between individual HDAC inhibitors [151]. In addition to enhancing interferon signaling, HDAC inhibition may also modulate immune checkpoint expression. Recent mechanistic evidence suggests that entinostat can upregulate PD-L1 in NKTCL through EGR1-dependent transcriptional activation. This observation highlights the epigenetic regulation of the PD-1/PD-L1 axis and provides additional biological context for combination strategies with immune checkpoint blockade [19]. Consistent with this biological rationale, the SCENT study evaluated the combination of sintilimab and chidamide in patients with relapsed/refractory NKTCL, achieving an ORR of 59.5% and a CR of 48.6%. The median DoR, PFS, and OS were 25.3, 23.2, and 32.9 months, respectively, with manageable hematologic adverse events as the predominant toxicities, indicating an acceptable safety profile for this combination regimen [38]. In addition, small real-world studies and clinical case series have suggested the feasibility of PD-1 inhibitor-based combinations with chidamide, such as the PCET regimen incorporating etoposide and thalidomide, with preliminary results supporting the potential of this strategy; however, validation in larger, well-designed studies remains necessary.

DNA methyltransferase inhibitors (DNMTi) represent another class of epigenetic modulators with therapeutic potential. By reversing methylation-mediated silencing of antigen presentation-related genes, inducing a “viral mimicry” effect, and activating type I interferon signaling, DNMTi provide an alternative and potent immunomodulatory approach for combination with immune checkpoint inhibitors [152]. A prospective study demonstrated that DNMTi priming followed by combination with sintilimab achieved an ORR of 66.7% and a CR rate of 47.6% in patients with relapsed/refractory NKTCL. Notably, several patients who had previously failed PD-1-based therapy regained clinical responses, suggesting that DNMTi priming may restore sensitivity to PD-1 blockade [153].

Collectively, combining epigenetic modulators with immune checkpoint inhibitors has a strong biological rationale and promising preliminary clinical activity in NKTCL. Further prospective studies are needed to confirm efficacy and optimize treatment scheduling and patient selection.

### 7.3. ICIs Combined with Metabolic Targeting Strategies

NKTCL exhibits characteristic features of EBV-driven metabolic reprogramming. LMP1 enhances aerobic glycolysis and promotes lactate accumulation through activation of NF-κB-dependent signaling, thereby driving tumor proliferation and contributing to an immunosuppressive metabolic milieu [52]. Beyond glycolytic rewiring, CPT1A, a key regulator of fatty acid oxidation, mediates succinylation of LDHA, a central glycolytic enzyme, and 14–3–3θ, a signaling adaptor involved in stress adaptation and cell survival, thereby highlighting metabolic crosstalk between fatty acid oxidation and glycolytic pathways and promoting tumor cell survival, metabolic adaptation, and chemoresistance [154,155]. In parallel, SLC1A1-driven glutamine addiction constitutes a critical metabolic vulnerability that sustains tumor growth while shaping the tumor immune microenvironment [63]. Collectively, these alterations establish a metabolically reprogrammed ecosystem characterized by enhanced glycolysis, lipid metabolic adaptation, and glutamine dependency [156].

Such metabolic remodeling not only supports tumor progression but also impairs effector T-cell fitness. Emerging evidence suggests that metabolic dysregulation may influence responses to PD-1/PD-L1 blockade. In particular, targeting glutamine metabolism has been shown to counteract CD8^+^ T-cell dysfunction in co-culture systems and to sensitize tumor cells to anti-PD-1 therapy, providing a mechanistic rationale for combining metabolic interventions with immune checkpoint inhibitors in NKTCL [63]. Additionally, the indoleamine 2,3-dioxygenase (IDO)–kynurenine pathway represents an immune–metabolic suppressive axis that may further limit the efficacy of PD-1 blockade [157]. Accordingly, integrating ICIs with metabolic targeting strategies represents a biologically grounded and promising avenue for therapeutic optimization.

Collectively, these emerging and expanded immunotherapeutic strategies do not currently rest on the same level of evidence in NKTCL. Some are supported by direct clinical data in NKTCL, whereas others remain supported mainly by preclinical studies or biologic extrapolation from other malignancies. A comparative summary of their current evidence level, biologic rationale, and major translational limitations is provided in Table 5.

## 8. Challenges and Future Directions

Despite substantial progress in multimodal immunotherapy, NKTCL remains biologically complex and therapeutically challenging, largely because durable responses continue to be constrained by the interplay among EBV-driven immune escape, suppressive tumor microenvironmental remodeling, and dynamic resistance mechanisms. Engineered cellular approaches face additional barriers, including antigen heterogeneity, limited persistence, and trafficking constraints.

Future advances will require a shift from empirical combination strategies toward biologically stratified intervention models. A key priority is to define multidimensional immune landscapes by integrating single-cell transcriptomics, spatial profiling, and longitudinal EBV-DNA dynamics, with the aim of identifying dominant exhaustion states and co-inhibitory receptor patterns within individual tumors. Such stratification may support the rational selection of matched bispecific or multi-checkpoint strategies rather than uniformly intensifying blockade. Translational validation in EBV-driven lymphoma models, followed by biomarker-guided prospective trials, will be essential for advancing more precise immunomodulation in NKTCL. Within this biomarker-guided framework, circulating EBV-DNA represents one of the more established biomarkers in NKTCL, with relevance to disease monitoring and prognostic/risk stratification, as reflected by its incorporation into the PINK-E model. Other, more established biomarkers, including tumor PD-L1 expression, PD-L1 structural rearrangements, and soluble or exosomal PD-L1, may support baseline stratification, response prediction, or longitudinal monitoring. By contrast, other tumor-intrinsic, immune-contextural, and metabolic features remain exploratory and are better regarded as candidate stratification markers. These biomarker categories are summarized in Table 3 as a framework for treatment stratification and disease monitoring in NKTCL.

Second, mechanism-driven combination immunotherapeutic strategies should be further developed, including the integration of PD-1 blockade with epigenetic modulation, metabolic targeting, antibody engagers, or engineered cellular therapies. In particular, future studies should move beyond empirical combinations toward rational sequencing strategies guided by dynamic immune-state profiling. Optimization of treatment sequencing, dosing, and therapeutic windows will be critical to overcoming both primary and acquired resistance.

Third, innovative immunotherapeutic platforms require disease-specific refinement. In particular, EBV-targeted mRNA vaccination represents a promising yet underdeveloped strategy in NKTCL. Overcoming profound tumor-associated immunosuppression will likely require optimization of antigen selection, delivery systems, and immune priming capacity. Comparative evaluation of multivalent EBV antigen constructs, engineering of nanoparticle-based delivery platforms designed for antigen-presenting cell targeting, and co-delivery of immune-stimulatory signals may enhance the durability of vaccine-induced T-cell responses. Integration or sequencing of therapeutic vaccination with checkpoint blockade may represent a promising strategy to enhance immune priming and help sustain T-cell function.

Overall, immunotherapy for NKTCL is moving beyond single-target inhibition toward more precise and integrated therapeutic strategies. Future progress will depend on a deeper understanding of disease biology, biomarker-guided patient selection, and rational clinical trial design, with the ultimate goal of achieving more durable clinical benefit.

## 9. Conclusions

In summary, immunotherapy for NKTCL is evolving from single-pathway blockade toward a more integrated and precision-oriented therapeutic paradigm. PD-1/PD-L1 inhibitors currently represent the cornerstone of treatment in NKTCL, while antibody-based therapies, engineered cellular approaches, EBV-specific immunotherapies, and emerging immune engagers are further broadening the therapeutic landscape through complementary mechanisms. In addition, epigenetic modulation, metabolic targeting, and immune strategies directed against EBV-associated antigens provide additional opportunities to remodel the tumor immune microenvironment and enhance sensitivity to immunotherapy.

These advances reflect a continuing shift toward more integrated and optimized immunotherapeutic strategies in NKTCL. Future progress will depend on more precise patient stratification, dynamic monitoring of immune-related biomarkers, and better-designed combination and sequencing strategies. As these approaches continue to be validated and refined, multimodal immunotherapy is likely to play an increasingly important role in NKTCL treatment and may ultimately lead to more durable clinical benefit for patients.

## Figures and Tables

**Table 2:** Major Resistance Mechanisms to Immunotherapy in NKTCL, with Corresponding Mechanism-Based Therapeutic Strategies and Representative Therapeutic Examples.

Resistance Category	Resistance Mechanism	Key Features	Mechanism-Based Therapeutic Strategies	Representative Therapeutic Examples
Primary resistance	Immunosuppressive TIME	EBV-driven immune suppression; enrichment of exhausted T cells, Tregs, and M2-like macrophages	Combinations to remodel the suppressive TIME; EBV-directed immunotherapy	PD-1/PD-L1-based combinations; EBV-CTL or EBV vaccines
	Baseline co-inhibitory signaling	PD-1, LAG-3, and TIM-3 co-expression	Dual/multi-checkpoint blockade	PD-1/PD-L1 blockade plus LAG-3/TIM-3 targeting
	Defective antigen presentation	HLA-I and β2-microglobulin downregulation	Epigenetic modulation; radiotherapy-based immune priming	Epigenetic therapy plus PD-1/PD-L1 blockade; radiotherapy plus PD-1/PD-L1 blockade
Acquired resistance	Adaptive immune escape	IFN-γ-driven secondary PD-L1 upregulation	ICI-based combination strategies	Chemotherapy, radiotherapy, or epigenetic therapy plus PD-1/PD-L1 blockade
	Therapy-associated metabolic reprogramming and physical barriers	Enhanced glycolysis and lactate accumulation, increased lipid metabolism, aberrant angiogenesis, and extracellular matrix deposition	Metabolic targeting; microenvironment-modulating strategies	Metabolic targeting plus PD-1/PD-L1 blockade; radiotherapy plus PD-1/PD-L1 blockade

Note: Some mechanisms may overlap between primary and acquired resistance; here they are categorized according to their predominant temporal and biological context. Abbreviations: EBV, Epstein–Barr virus; EBV-CTL, Epstein–Barr virus-specific cytotoxic T lymphocyte; HLA-I, human leukocyte antigen class I; ICI, immune checkpoint inhibitor; IFN-γ, interferon-γ; LAG-3, lymphocyte activation gene-3; NKTCL, natural killer/T-cell lymphoma; PD-1, programmed cell death protein 1; PD-L1, programmed death-ligand 1; TIME, tumor immune microenvironment; TIM-3, T-cell immunoglobulin and mucin-domain containing-3; Treg, regulatory T cell.

**Table 3:** Biomarker-informed framework for immunotherapy stratification and monitoring in NKTCL.

Category	Biomarker or Biologic Feature	Detection Method	Potential Utility	Key Limitations	Reference
More established biomarkers	Circulating EBV-DNA	Plasma PCR-based assay	Disease monitoring; response assessment; dynamic risk assessment; prognostic stratification	Methodologic and threshold variability; limited immune specificity	[56,57,58,59]
	Tumor PD-L1 expression	Tissue IHC	Baseline stratification; immune-evasive phenotype	Dynamic expression; variability in antibodies, scoring systems, and cutoffs; limited predictive standardization	[13,16,20,21]
	PD-L1 structural rearrangements	Tumor genomic testing	Potential predictor of response to PD-1 blockade	Low prevalence; limited prospective validation	[60]
	Soluble or exosomal PD-L1	Serum/plasma assay	Blood-based assessment of disease burden or immune-evasive states; longitudinal immune-related monitoring	Non-standardized assays; variable origin and kinetics	[13,61]
Exploratory stratification features	HLA-I/β2-microglobulin loss or antigen-presentation defects	Tissue IHC and/or genomic profiling	Impaired antigen presentation; potentially reduced benefit from T-cell-dependent immunotherapies	Predictive value not yet validated	[46,62]
	PD-1/LAG-3/TIM-3 co-expression or exhaustion signatures	Multiplex IHC, flow cytometry, or single-cell profiling	Deep exhaustion states; rationale for dual/multi-checkpoint strategies	Complex assays; heterogeneity; no standardized cutoffs	[44,47,48,49,50,51]
	Macrophage-rich phenotype/EBV^+^ macrophage enrichment	Spatial transcriptomics or multiplex tissue profiling	Suppressive TIME state; rationale for TIME-remodeling approaches	Mainly translational evidence; limited clinical standardization	[45]
	Immune-excluded spatial phenotype	Spatial transcriptomics/multiplex imaging	Impaired effector-cell infiltration; rationale for immune-priming strategies	Advanced platforms required; non-standardized classification	[45,55]
	Glycolytic, lipid-remodeled, or glutamine-dependent metabolic states (e.g., FABP5, SLC1A1)	Transcriptomic, metabolic, or tissue expression profiling	Immunometabolic suppression; rationale for metabolic co-targeting	Exploratory only; no validated clinical threshold	[52,53,63]

Abbreviations: EBV, Epstein–Barr virus; FABP5, fatty acid-binding protein 5; HLA-I, human leukocyte antigen class I; IHC, immunohistochemistry; LAG-3, lymphocyte activation gene-3; PCR, polymerase chain reaction; PD-1, programmed cell death protein 1; PD-L1, programmed death-ligand 1; SLC1A1, solute carrier family 1 member 1; TIME, tumor immune microenvironment; TIM-3, T-cell immunoglobulin and mucin-domain containing-3.

**Table 5:** Emerging and expanded immunotherapeutic strategies in NKTCL: evidence level, rationale, and limitations.

Strategy	Examples	Clinical Evidence in NKTCL	Preclinical Evidence in NKTCL	Other-Tumor Support	Rationale in NKTCL	Major Limitations
Multi-checkpoint blockade/PD-1-based bispecific ICIs	LAG-3/TIM-3 targeting; tebotelimab; EMB-02; lomvastomig; cadonilimab; rilvegostomig	No dedicated trial	Not clearly established	Yes	Multi-checkpoint co-expression; deep T-cell exhaustion	Mainly extrapolated; clinical relevance in NKTCL unproven
Antibody-based targeted therapies	Daratumumab; isatuximab; brentuximab vedotin; CD70-targeted ADCs	Direct clinical evidence exists for daratumumab and brentuximab vedotin, whereas evidence for isatuximab and CD70-targeted ADCs remains mainly extrapolated	Limited/target-dependent	Yes	Surface antigen targeting; ADCC/CDC or payload delivery	Antigen heterogeneity; limited durability; off-tumor effects
CAR-T therapy	CD38-, CD56-, CD7-, CD30-, EBV-LMP1-directed CAR-T	No mature subtype-specific efficacy data	Direct preclinical evidence is available for selected targets, whereas evidence for others remains more limited	Yes	Targeting tumor-associated or EBV-related antigens	Antigen heterogeneity; fratricide/off-tumor toxicity; suppressive TIME; poor T-cell fitness
EBV-CTL therapy	Autologous or third-party EBV-specific CTLs	Yes	Direct NKTCL-specific preclinical evidence is limited, with broader support deriving from other EBV-associated malignancies	Yes	Multi-epitope targeting of EBV latent antigens	Manufacturing complexity; HLA restriction; limited durability
CAR-NKT and other engineered cellular platforms	CAR-NKT; γδ T-cell-based approaches	No direct data	No direct NKTCL-specific data	Yes	Lower GVHD risk, off-the-shelf potential, and possible activity in settings with antigen heterogeneity	Very limited NKTCL-specific data; expansion and persistence challenges
T-cell engagers (TCEs)	CD38 × CD3; CD70 × CD3	No direct data	No direct NKTCL-specific data	Yes	Redirecting T cells to NKTCL-associated antigens	No dedicated NKTCL studies; antigen heterogeneity; uncertain durability
NK-cell engagers (NKCEs)	AFM13; BiKEs; TriKEs	No direct data	No direct NKTCL-specific data	Yes	Harnessing NK-cell cytotoxicity against relevant targets	Largely extrapolated; no direct efficacy data in NKTCL
Therapeutic EBV vaccines	EBNA1/LMP2-based vaccines; mRNA-based EBV vaccines	Only early or basket clinical exploration including NKTCL has been reported	No clearly established NKTCL-specific preclinical evidence; support remains mainly indirect	Yes	Restoring EBV-specific immunity in an EBV-driven disease	Very limited NKTCL-specific evidence; immune suppression may blunt efficacy
Multimodal combinations	ICIs + radiotherapy; ICIs + epigenetic therapy; ICIs + metabolic targeting	Yes (radiotherapy/epigenetic); limited for metabolic targeting	Yes	Yes	Overcoming immune exclusion, antigen-presentation defects, and immunometabolic suppression	Limited comparative data; sequencing and patient selection remain unclear

Abbreviations: ADC, antibody-drug conjugate; ADCC, antibody-dependent cellular cytotoxicity; CAR-T, chimeric antigen receptor T-cell; CAR-NKT, chimeric antigen receptor natural killer T-cell; CDC, complement-dependent cytotoxicity; CTL, cytotoxic T lymphocyte; EBV, Epstein–Barr virus; GVHD, graft-versus-host disease; ICI, immune checkpoint inhibitor; NKTCL, natural killer/T-cell lymphoma; NKCE, natural killer cell engager; TCE, T-cell engager; TIME, tumor immune microenvironment.

## Data Availability

No new data were created or analyzed in this study. Data sharing is not applicable to this article.

## Abbreviations

The following abbreviations are used in this manuscript:

ADCC	antibody-dependent cellular cytotoxicity
ADC	antibody–drug conjugate
AE	adverse event
allo-HSCT	allogeneic hematopoietic stem cell transplantation
AML	acute myeloid leukemia
ATP	adenosine triphosphate
B-NHL	B-cell non-Hodgkin lymphoma
BV	brentuximab vedotin
CAR	chimeric antigen receptor
CAR-T cells	chimeric antigen receptor T cells
CAR-NKT cells	chimeric antigen receptor natural killer T cells
CDC	complement-dependent cytotoxicity
CR	complete response
CRR	complete response rate
CRS	cytokine release syndrome
CRT	calreticulin
CTLA-4	cytotoxic T-lymphocyte-associated protein 4
DAMPs	damage-associated molecular patterns
DC	dendritic cell
DNMTi	DNA methyltransferase inhibitor
DoR	duration of response
EBNA1	Epstein–Barr virus nuclear antigen 1
EBV	Epstein–Barr virus
EBV-CTLs	Epstein–Barr virus-specific cytotoxic T lymphocytes
EGR1	Early Growth Response 1
ENKTL	extranodal NK/T-cell lymphoma
FABP5	fatty acid-binding protein 5
Gln	glutamine
GVHD	graft-versus-host disease
HDACi	histone deacetylase inhibitor
HLA-I	human leukocyte antigen class I
HLH	hemophagocytic lymphohistiocytosis
HMGB1	high-mobility group box 1
ICANS	immune effector cell-associated neurotoxicity syndrome
ICD	immunogenic cell death
ICIs	immune checkpoint inhibitors
IDO	indoleamine 2,3-dioxygenase
IFN-I	type I interferon
IFN-γ	interferon-γ
IL	interleukin
irAEs	immune-related adverse events
Kyn	kynurenine
LAG-3	lymphocyte activation gene-3
LMP1	latent membrane protein 1
LMP2A	latent membrane protein 2A
mOS	median overall survival
mPFS	median progression-free survival
MDS	myelodysplastic syndromes
MHC	major histocompatibility complex
N/A	not available
NK	natural killer
NKCEs	natural killer cell engagers
NKTCL	natural killer/T-cell lymphoma
NHL	non-Hodgkin lymphoma
NR	not reported
NSCLC	non-small cell lung cancer
ORR	objective response rate
OS	overall survival
PCR	polymerase chain reaction
PD-1	programmed cell death protein 1
PD-L1	programmed death-ligand 1
PFS	progression-free survival
PR	partial response
PTCL	peripheral T-cell lymphoma
R/R	relapsed or refractory
SAEs	serious adverse events
SD	stable disease
SLC1A1	solute carrier family 1 member 1
TCEs	T-cell engagers
TIGIT	T-cell immunoreceptor with Ig and ITIM domains
TIME	tumor immune microenvironment
TIM-3	T-cell immunoglobulin and mucin-domain containing-3
TLS	tumor lysis syndrome
TRAFs	tumor necrosis factor receptor-associated factors
TRD	treatment-related death
Treg	regulatory T cell
Trp	tryptophan
yr	year(s)
mo	month(s)
